# Electroencephalographic dynamics of etomidate‐induced loss of consciousness

**DOI:** 10.1186/s12871-021-01308-7

**Published:** 2021-04-08

**Authors:** Lei Zhang, Shunqin Fan, Jiawei Zhang, Kun Fang, Lei Wang, Yuanyuan Cao, Lijian Chen, Xuesheng Liu, Erwei Gu

**Affiliations:** 1 Department of Anesthesiology, The First Affiliated Hospital of Anhui Medical University, No. 218 Jixi road, Anhui province 230022 Hefei, China; 2 The First Medical College of Anhui Medical University, 230032 Hefei, China; 3 Key Laboratory of Anesthesiology and Perioperative Medicine of Anhui Higher Education Institutes, Anhui Medical University, Hefei, China

**Keywords:** EEG, Etomidate, LOC, General anesthesia

## Abstract

**Background:**

Highly structured electroencephalography (EEG) oscillations can occur in adults during etomidate-induced general anesthesia, but the link between these two phenomena is poorly understood. Therefore, in the present study, we investigated the electroencephalogram dynamics of etomidate-induced loss of consciousness (LOC) in order to understand the neurological mechanism of etomidate-induced LOC.

**Methods:**

This study is a prospective observational study. Etomidate-induced anesthesia was performed on eligible patients undergoing elective surgery. We analyzed EEG data from 20 patients who received etomidate for the induction of general anesthesia. We used power spectra and coherence methods to process and analyze the EEG data. Our study was based on 4-channel EEG recordings.

**Results:**

Compared with the baseline (awake period), etomidate induced an increase in power in delta, theta, alpha and beta waves during LOC. Compared with the awake period, the delta-wave (1–4 Hz), alpha-wave(8–13 Hz), and theta-wave(4–8 Hz) coherence increased significantly during LOC, while the slow-wave (< 1 Hz) coherence decreased. However, the delta wave (1.0–4.0 Hz) during etomidate-induced LOC was more coherent than during the awake period (1.86–3.17 Hz, two-group test for coherence, *p <* 0.001).

**Conclusions:**

The neural circuit mechanism of etomidate-induced LOC is closely related to the induction of oscillation in delta, theta, alpha and beta waves and the enhancement of delta-wave coherence.

**Trial registration:**

ChiCTR1800017110

## Background

Etomidate is a non-barbiturate intravenous anesthetic that has a rapid onset of action and induces a stable, quiet, comfortable, and non-excitable transition during the induction period. Enhancement of γ-aminobutyric acid A receptor (GABAAR) activity is considered to be the primary mechanism mediating etomidate-induced anesthesia [1, 2]. Etomidate has little effect on the cardiovascular and respiratory systems. Based on these characteristics, etomidate is widely used in the induction of anesthesia in patients with impaired hemodynamics, such as in the elderly and those with cardiovascular disease or critical illness [3, 4]. General anesthetics are well described at the molecular level and cellular pharmacological level, but the neural circuit mechanisms that cause LOC remain unclear [5, 6]. Electroencephalography (EEG) is considered to be the most direct indicator of central nervous system activity [7, 8]. The quantitative evaluation of the depth of anesthesia by EEG has made an important contribution to the practice of clinical anesthesia [7]. A recent study reported that in general anesthesia, the effect of propofol on neuronal data recorded in the frontal cortex, and the study found that frontal-lobe electrical activity is the precursor to a LOC [9].

Etomidate-induced general anesthesia produces dynamic changes in EEG data[10], but the characteristics of these changes have not been well elucidated. Furthermore, the relationship between etomidate-induced LOC and characteristic changes in EEG data is not well understood. Kuizenga et al.[10] studied the amplitude of the specified band, spectral edge frequency 95 % (SEF95) and bispectral index (BIS), to explore the relationship between EEG effects and the moment of LOC.Our research mainly used spectral and coherence analysis to clarify the relationship between etomidate-induced LOC and the characteristic changes in EEG dynamics.

## Methods

This study followed the Declaration of Helsinki and was approved by the Ethics Committee of the First Affiliated Hospital of Anhui Medical University. Standard monitoring techniques (noninvasive blood pressure, electrocardiography [ECG], and pulse oximetry) were applied. Hemodynamic variables were recorded every 60 s. Written informed consent was obtained from the included patients, who were between 18 and 65 years old (American Society of Anesthesiologists (ASA): class I to II; No gender limit) and required general anesthesia. Exclusion criteria were as follows: pregnancy, hearing impairments, mental disorders, or taking drugs that may interfere with the accuracy of EEG recordings. Myoclonus often occurs during the induction of etomidate anesthesia [11, 12]. The patient’s EEG data was not included in the data analysis if myoclonus occurred clinically.

**Fig. 1:**
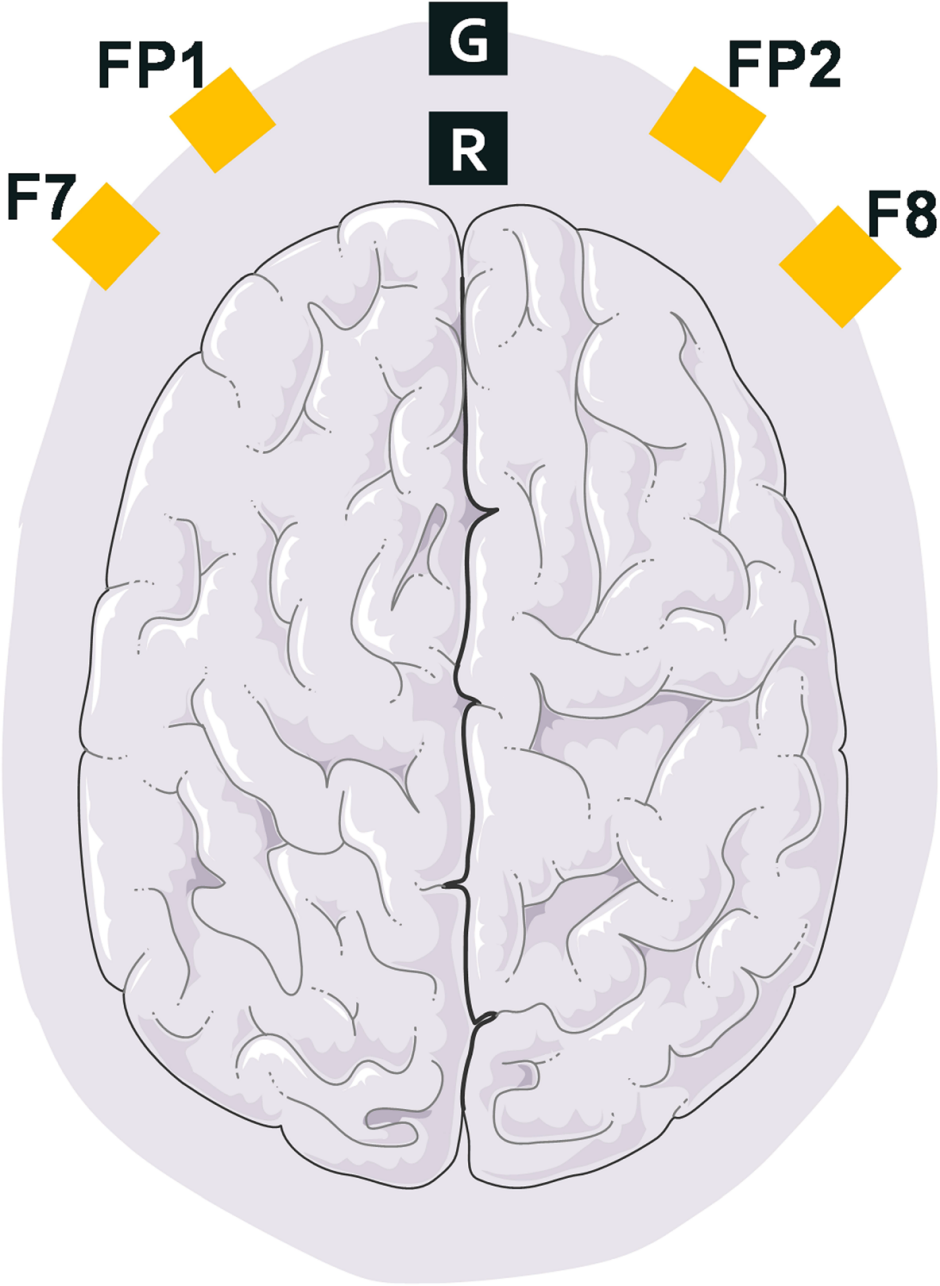
The channel position and the two bipolar frontal channels: F7 and F8, which we used for coherence analysis

We used a four-channel Sedline brain function monitor (Masimo,Irvine, CA,USA) to record the frontal-lobe EEG data. EEG data was recorded in patients undergoing elective surgery from a baseline of 3 min (awake and quiet with their eyes closed) to 3 min of etomidate-induced LOC. Etomidate (0.06 mg/kg/min) was the only anesthetic used [10]. The EEG data was recorded with a preamplifier bandwidth of 0.5–92 Hz, a sampling rate of 178 Hz, 16 bits. The standard Sedline-Sedtrace electrode array records were from electrodes located roughly at positions Fp1, Fp2, F7, and F8, with the ground electrode at Fpz and the reference electrode at roughly 1 cm above Fpz (Fig. 1). The electrode impedance was less than 5 kΩ in each channel. The time recorded in the case report form was required to match the time on the EEG recorder in order to mark key events (e.g., induction start, LOC) during the analysis.

### Criteria for the LOC

At present, in the induction of clinical anesthesia, the determination of the LOC is assessed by aimless movements after harmful stimulation [13]. In the present study, the auditory stimulation assessment [14, 15] was supplemented by the disappearance of the eyelash reflex to confirm the LOC. Before inducing anesthesia, we instructed patients not to open or move their eyes.

### Data preprocessing

A researcher with experience in reading electroencephalograms manually browsed the EEG data of each patient to manually remove artifacts. The investigator used the recorded information in the case report form to select the appropriately timed EEG data segment. For each case, the EEG segment representing 60 s of consciousness and closed eyes was carefully selected during the perioperative period, as was the EEG segment corresponding to 60 s after the LOC, for data analysis.

### Spectral analysis

**Fig. 2:**
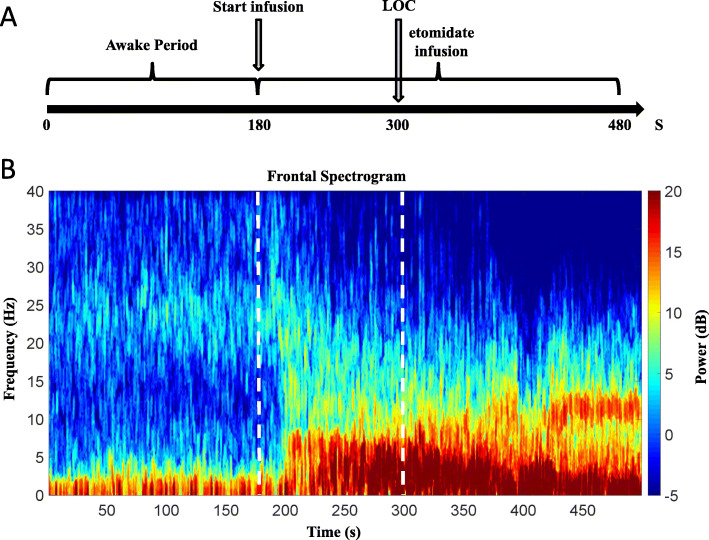
Time-frequency analysis of EEG data between the awake period and etomidate-induced LOC. **a** During the awake period before induction of etomidate (*n* = 20), slow-wave (< 1.0 Hz) and delta-wave (1.0–4.0 Hz) oscillations were mainly present. **b** Etomidate-induced LOC (*n* = 20) yielded an increase in the power of slow waves (< 1.0 Hz), delta waves (1.0–4.0 Hz), theta waves (4.0–8.0 Hz), and alpha waves (8.0–13.0 Hz). **c** Spectrograms of the awake period. Compared with those during the awake period, the powers of the slow wave (< 1.0 Hz), delta wave (1.0–4.0 Hz), theta wave (4.0–8.0 Hz), and alpha wave (8.0–13.0 Hz) during the etomidate-induced LOC were significantly increased (C: 0–22.97 Hz, 27.28–40.00 Hz; *p* < 0.001, two-group test for spectra). Median spectra presented with 95 % jackknife CIs. Horizontal solid black lines represent the frequency ranges at which there were significant differences

**Fig. 3:**
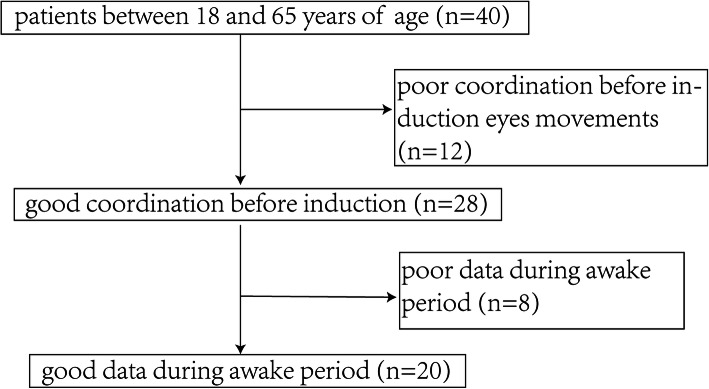
Frontal-lobe EEG spectrograms in the study of etomidate-induced LOC. **a** In the etomidate study, the main events are marked on the timeline. **b** During the study, each patient was instructed to keep their eyes closed and without motion for a period of 3 min before etomidate was administered to induce general anesthesia. The frequency is plotted on the y-axis and time is plotted on the x-axis. The energy or power in the signal is represented by color. As the induction of etomidate began, the slow-wave, delta-wave, and theta-wave oscillations increased. After LOC, the alpha wave oscillation increased

The power spectrum quantifies the energy of each frequency of the EEG. The spectrogram was computed using the multitaper method achieved in the Chronux toolbox in MATLAB[16]. The group-level spectrogram computed by taking the median of spectrograms of all patients (Fig. 2 a/b). The spectrum of the selected EEG epochs (EEG epoching is a procedure in which specific time-windows are extracted from the continuous EEG signal) was also calculated by us. Then, for all epochs, the resulting power spectra was averaged, and by way of multitaper-based jackknife techniques [16], 95 % confidence intervals (CIs) were computed. Parameters for spectral analysis are as follows: window length T = 2 s with a 1.95 s overlap; time-bandwidth product of TW = 3 (Approximately 2WT Slepian functions fit on this Time-Frequency tile. Since T, W are input parameters, we can easily control the resolution element in the Time Frequency plane using Slepians); number of tapers, *K* = 5; and spectral resolution = 3 Hz (Fig. 3).

### Coherence analysis

Coherence graphs are coherent time-varying versions, which are estimated using continuous windows of EEG data. Between two signals, x and y, the coherence Cxy (f) function, is determined as follows:

$$\text{C}\text{x}\text{y}\left(\text{f}\right)=\frac{ | \text{S}\text{x}\text{y}\left(\text{f}\right) | }{\surd \text{S}\text{x}\text{x}\left(\text{f}\right)\text{S}\text{y}\text{y}\left(\text{f}\right)},$$

Sxy (f) is the cross-spectrum between the signals x (t) and y (t), Sxx (f) is the power spectrum of the signal x (t), and Syy (f) is the power spectrum of the signal y (t) [16]. In order to acquire the appraised coherence, based on the Chronux toolbox in MATLAB, the coherence was computed between F7 and F8, the two frontal electrodes[17]. By taking the median across subjects, the group-level coherograms were computed. The coherence for selected EEG epochs was also calculated. The resulting coherence estimates were averaged for all epochs, and by way of multitaper-based jackknife techniques,95 % CIs were computed [16]. Parameters for the coherence analysis were similar to spectral analysis and spectral resolution of 2 W = 3 Hz.

### Statistical analysis

So as to compare spectral and coherence estimates between groups, we utilized jackknife-based methods [16], the two-group test for spectra, and the two-group test for coherence, as performed in the Chronux toolbox routine [18]. This method takes into account the frequency spectrum and the basic spectral resolution of the coherence estimation, and only when the difference occurs at a continuous frequency on a frequency band wider than the spectral resolution of 2 W, the difference is considered significant. To be specific, for frequencies f > 2 W, the negative assumption was rejected only if the test statistic surpassed the significance threshold over a contiguous frequency range ≥ 2 W. For frequencies 0 ≤ f ≤ 2 W, in order to illustrate the capabilities of multitaper spectral estimation when the frequency is near zero, the negative assumption was rejected only if the test statistic surpassed the significance threshold over a contiguous frequency range from 0 to max (f,W) ≤ 2 W. A significance threshold of *p* < 0.001 was confirmed for comparisons between groups.

## Results

### Case selection

**Fig. 4:**
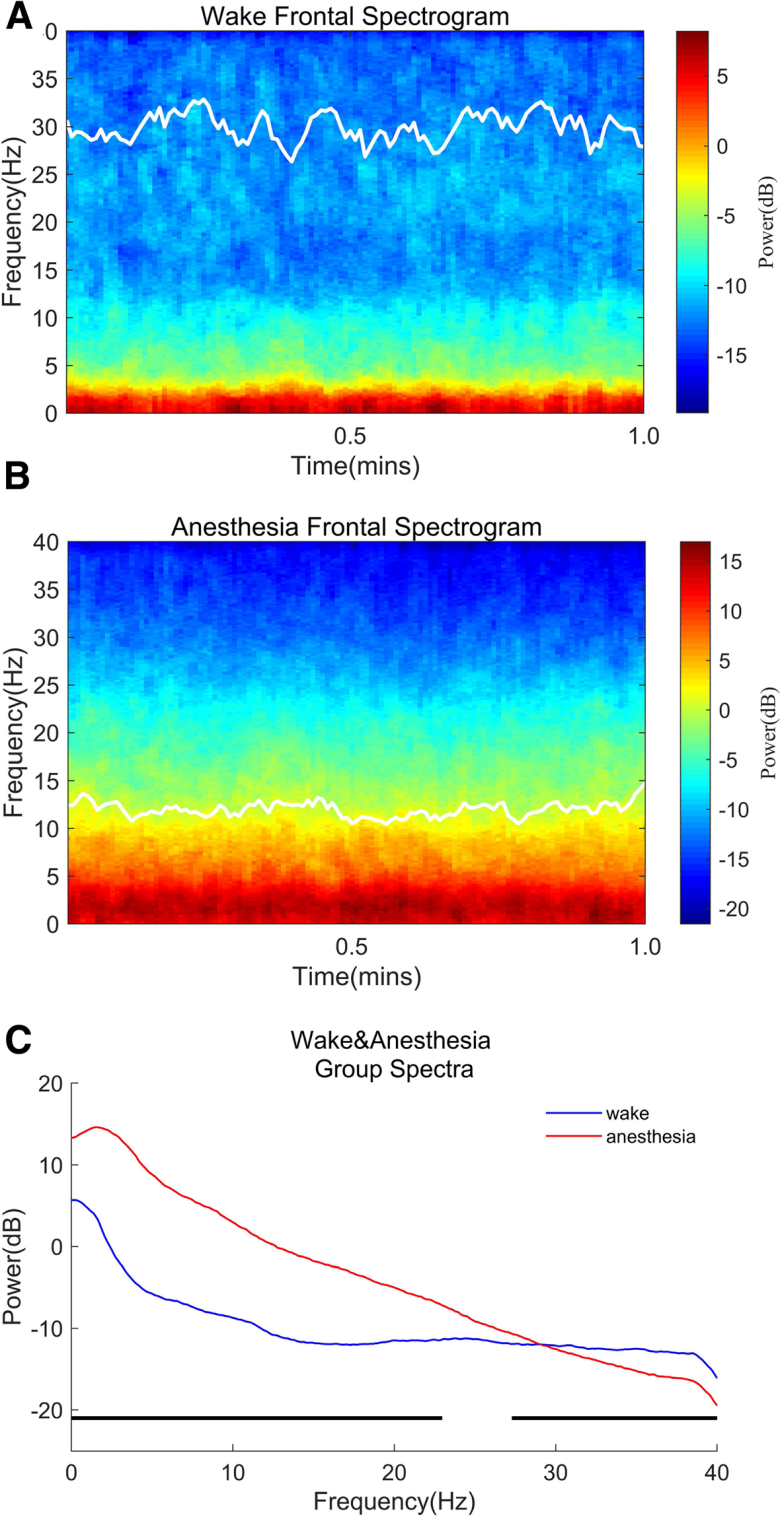
Case selection. Our study initially included 40 individuals between 18 and 65 years old. We excluded 12 patients because of restlessness or other non-cooperative behaviors before anesthesia. We then examined the EEG data of the remaining 28 patients and excluded 8 patients due to poor data quality (Poor electrode contact and other reasons). Ultimately, we analyzed the EEG data from 20 patients

Our study initially included 40 individuals between 18 and 65 years old. Of these, we excluded patients who had poor cooperation before anesthesia (*n* = 12). Then we imported the EEG data of the remaining patients (*n* = 28) into EEGLAB and excluded patients (*n* = 8) due to poor electrode contact (Fig. 4). Table 1 shows the basic demographic and clinical characteristics of the included cases.

**Table 1:** Basic features information of case objects

	Etomidate (*n* = 20)
Sex (male/%)	8 (40)
Age (yr), mean (**±** SD)	36 (10)
Weight (kg), mean (± SD)	61 (9)
Height (cm), mean (± SD)	164 (7)
Time of LOC (s), mean (± SD)	168 (7)

### Etomidate power‐spectra analysis

We observed the EEG spectra during both the awake period before etomidate administration and during etomidate-induced LOC. The two time-frequency diagrams were continuous in time, and the EEG power changed significantly over time. The awake period and the period corresponding to the etomidate-induced LOC were dominated by slow-wave (0.1–1.0 Hz) and delta-wave (1.0–4.0 Hz) oscillations. And the period of LOC also showed the theta-wave (4.0–8.0 Hz) and alpha-wave (8.0–13.0 Hz) oscillations. Next, we performed time-frequency analysis of EEG data in the two periods and found that after etomidate-induced LOC, the oscillation in delta, theta, alpha and beta waves increased. We also observed that the two periods were clearly different in time and frequency between 0 and 22.97 Hz and 27.28–40.00 Hz (Fig. 2).

### Etomidate coherence analysis

**Fig. 5:**
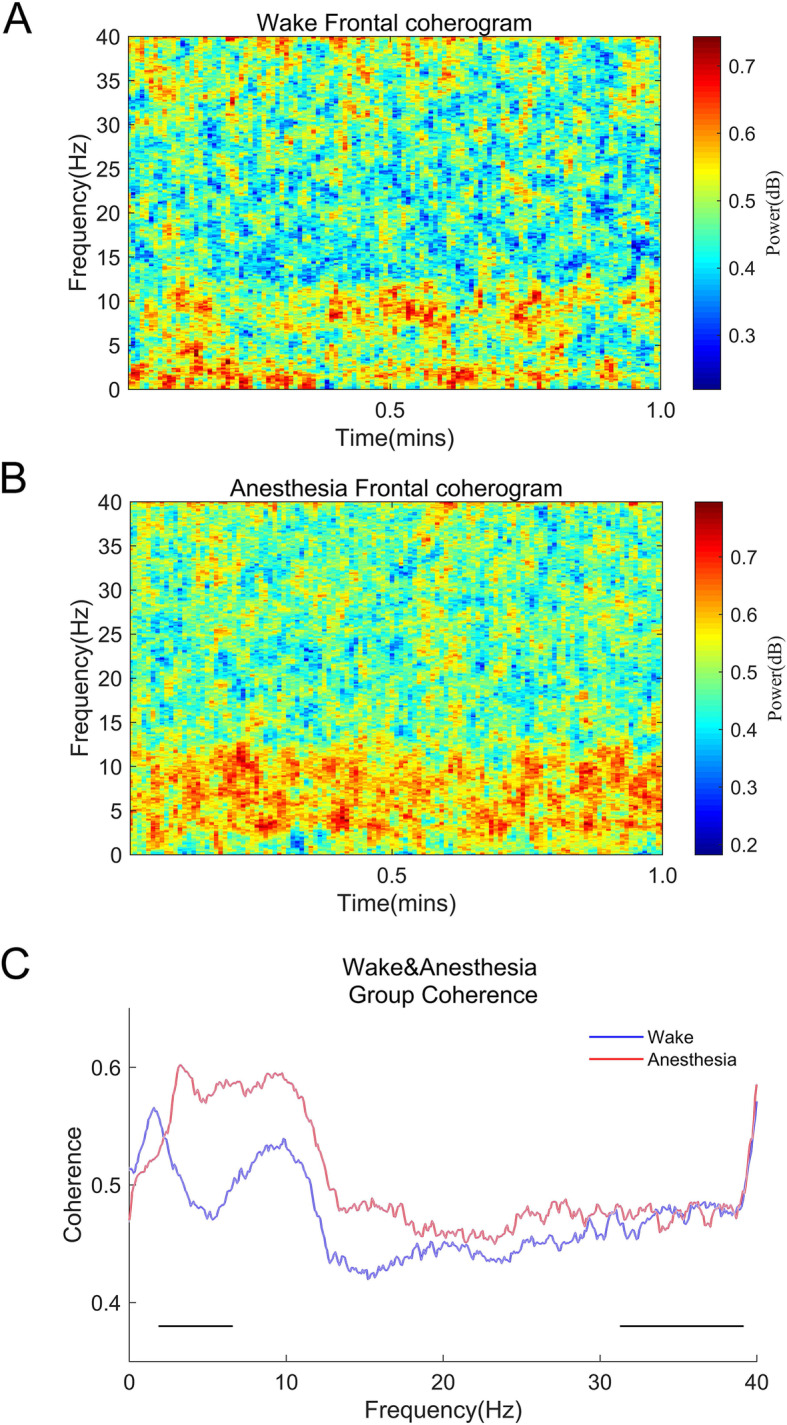
Coherence analysis between the awake period and the period during etomidate-induced LOC. **a** The coherence analysis of the awake period (*n* = 20) showed the coherence spectrum in terms of the slow wave (< 1.0 Hz), delta wave (1.0–4.0 Hz), and alpha wave (8.0–13.0 Hz). **b** The coherence analysis during etomidate-induced LOC (*n* = 20) shows the coherence spectra in terms of the delta wave (1.0–4.0 Hz), theta wave (4.0–8.0 Hz), and the alpha wave (8.0–13.0 Hz). During the awake period, the coherence of slow waves (< 1.0 Hz) was decreased. **c** The coherence spectra of the awake period and the period during induced LOC. The alpha wave (8.0–13.0 Hz) coherence is similar in both periods. However, the delta wave (1.0–4.0 Hz) during etomidate-induced LOC was more coherent than during the awake period (1.86–3.17 Hz, two-group test for coherence, *p* < 0.001). Median coherence is presented with 95 % jackknife CIs. Horizontal solid black lines represent frequency ranges at which there was significant difference. The bandpass filter allows frequency components to pass within a certain frequency range but attenuates the frequency components in other ranges to a very low level rather than cutting off frequencies outside the range at a certain frequency point; therefore, at 40 Hz, the graph shows that the coherence is uniform and rising

We next analyzed the similarities and differences in the correlation patterns between the awake period and the period during etomidate-induced LOC. The period of awake showed the coherence spectrum in terms of the slow wave (< 1.0 Hz), delta wave (1.0–4.0 Hz), and alpha wave (8.0–13.0 Hz). The period of etomidate-induced LOC shows the coherence spectra in terms of the delta wave (1.0–4.0 Hz), theta wave (4.0–8.0 Hz), and the alpha wave (8.0–13.0 Hz). During the awake period, the coherence of slow waves (< 1.0 Hz) was decreased. We also observed that there was a significant difference in the coherence between the waking period and the period of etomidate-induced LOC in the 1.86–3.17 Hz band (Fig. 5).

## Discussion

In the present study, we analyzed the characteristic changes in the EEG bands before and during etomidate-induced anesthesia via spectral analysis and coherence analysis. From the awake period to the LOC period, etomidate general anesthesia induced an increase in oscillation in each frequency band within the EEG data. Additionally, the coherence of the EEG signal in the theta wave and alpha wave was obviously enhanced, when etomidate induced LOC; and the delta wave also showed obvious coherence.

Etomidate exerts its anesthetic actions through potentiation of GABAARs containing β2 and β3 subunits. It has recently been shown that the β2 subunit contributes to the sedative properties of etomidate, whereas the β3 subunit is responsible for its anesthetic properties [19, 20]. In our present study, etomidate induced anesthesia and enhanced theta-wave oscillations. In our coherence analysis, etomidate produced strong coherence in the theta wave; interestingly, β3-subunit-containing receptors play an important role in theta-wave oscillations. A previous study analyzed the effects of etomidate on rhythmic population activity by recording local field potentials (LFPs) [21]. In slices, which are derived from wild-type mice, etomidate (200 nM) amplified the oscillatory population activity in the theta-frequency band, but this effect was not seen in slices, which derived from β3-knockin mice, and this phenomenon was also observed in vivo [22]. These findings indicate that the neuronal mechanism of etomidate-induced LOC is closely related to the β3 subunit of GABAAR functional activity. Furthermore, previous evidence has shown that the overall effect of etomidate reflects a balance between enhancement and inhibition produced by GABAARs containing β2 and β3 subunits [21]. Etomidate enhances theta oscillations by acting on β3-containing GABAA receptors but depresses these oscillations via β2-subunit-containing receptors. In our present study of etomidate-induced LOC, we systematically observed the enhancement of EEG theta oscillations. However, which receptors participate and play a role is not known in our research, and further observation and research are needed.

In the present study, etomidate induced LOC and changed the EEG theta rhythm. Some studies have indicated that hippocampal interactions with the prefrontal cortex, another significant memory-associated structure, are coordinated by theta-rhythm plasticity [23, 24]. Amnesia is a significant symptom of general anesthesia. Several different brain areas participate in memory formation, including the prefrontal cortex, amygdala, and hippocampus. Anesthesia may be associated with memory impairment, as has been indicated by many studies in the hippocampus [25, 26], and evidence suggests that different frequency oscillations are related to changes in information coding and synaptic weights [27]. Etomidate-induced amnesia arises by means of GABAAR modulation, which highly depends on α-5 subunit modulation; this process likely occurs principally within the hippocampus [28, 29]. Anesthetics may disturb the greatly organized rhythmic activity patterns that are thought to be indispensable for hippocampal learning [30]. For instance, changes in theta frequency and theta power may contribute to amnesia by means of anesthetic actions on a variety of molecular targets [31]. Theta rhythms act as internal clocks that synchronize large networks and serve as a reference mechanism for internal and external synchronization, which is greatly coherent throughout the medial temporal lobe. These findings suggest that etomidate-induced LOC may be related to the changes in hippocampal theta rhythms. Collectively, these potential associations have led us to hypothesize that etomidate-induced theta oscillations may be an indicator of a functional disconnection between the hippocampus and cerebral cortex.

The EEG data analyzed in this study were all derived from the frontal 4-channel pathway, so our analysis was unable to assess other reported cortical kinetic connectivity associated with anesthetic-induced LOC. The results have been obtained with frontal electrodes only as a study limitation, so our observations need to be further validated in future high-density EEG studies[32]. At present, exploring the relationship between the neural circuit mechanism of etomidate-induced LOC and the characteristic changes of EEG can only be carried out in animal experimental models. Therefore, the clinical observed ‘the connection’ still needs to be verified by animal models.

According to our analysis and discussion, from the awake period to the LOC, the neural circuit mechanism of etomidate-induced LOC is closely related to the enhancement of delta-wave, alpha-wave, and theta-wave coherence. The characteristic transformation of the EEG that we described can be calculated and displayed in real time, providing a good reference for the monitoring of the depth of anesthesia and the evaluation of the level of sedation and consciousness by an anesthesiologist.

## Conclusions

The neural circuit mechanism of etomidate-induced LOC is closely related to the induction of oscillation in each EEG band and is closely related to the enhancement of delta-wave coherence. Through the analysis of EEG, our study elaborates the neural circuit mechanism related to the LOC induced by etomidate, which provides a feasible direction for subsequent clinical and basic studies.

## Data Availability

All data generated or analyzed during this study are included in this published article.

## Abbreviations

EEG: Electroencephalography
LOC: Loss of Consciousness
SEF95: Spectral Edge Frequency 95 %
BIS: Bispectral Index
GABAAR: γ-aminobutyric Acid A Receptor
ECG: Electrocardiography
ASA: American Society of Anesthesiologists
LFPs: Local Field Potentials
